# Arterial embolization of an extrapleural hematoma from a dislocated fracture of the lumbar spine: a case report

**DOI:** 10.1186/1757-7241-17-27

**Published:** 2009-06-09

**Authors:** Seiji Morita, Tomoatsu Tsuji, Tomokazu Fukushima, Takeshi Yamagiwa, Hiroyuki Otsuka, Sadaki Inokuchi

**Affiliations:** 1 Tokai University School of Medicine, Department of Emergency and Critical Care Medicine, 143 Shimokasuya Isehara-city, Kanagawa, 2591193, Japan

## Abstract

**Background:**

We present a report of a blunt-trauma patient who developed an atypical extrapleural hematoma with hemodynamic instability following a dislocation fracture of the first lumbar vertebra. We successfully treated her with arterial embolization (AE) of the lumbar and intercostal arteries.

**Case report:**

The patient, a 74-year-old woman, was injured in a traffic accident. At the scene of the accident, she was found to be alert, and her hemodynamic condition was stable. She arrived at our hospital complaining of lumbago. A thoracoabdominal computed tomography (CT) scan with contrast enhancement showed a dislocation fracture of the first lumbar vertebra along with paravertebral and retroperitoneal hematomas. Therefore, we managed the patient conservatively with bed rest. However, 3 h after admission, her blood pressure suddenly decreased. A repeated thoracoabdominal CT scan showed enlargement of the right retroperitoneal hematoma with extravasation of the contrast medium into the right extrapleural space. Angiography was immediately performed, showing extravasation of the contrast media from the right intercostal (Th12) and lumbar arteries (L1). After arterial embolization (AE) with gelatin-sponge particles, extravasation of the contrast medium ceased, and the patient's hemodynamic condition stabilized without massive fluid resuscitation.

**Conclusion:**

The extrapleural hematoma reduced in size after AE, and almost disappeared on the 14^th ^day of hospitalization. The lumbar spinal fracture was successfully repaired on day 16, and the patient was kept in the hospital to recuperate. We believe that AE is effective for the management of intractable bleeding following fractures of the spine.

## Introduction

An extrapleural hematoma (EH) is defined as the accumulation of blood in the extrapleural space [1]. A typical radiological finding of EH is a D-shaped opacity with its base against the chest wall. EH has been reported to frequently occur after blunt trauma causing tears or rupture of the blood vessels in the chest wall and fractures of the sternum and ribs. In contrast, life-threatening hematoma following fractures of the spine is uncommon. There have been few reports on the treatment of this condition with arterial embolization (AE), and AE is not an established therapeutic approach for this condition [2].

We present the report of a blunt-trauma patient who developed an atypical EH with hemodynamic instability following a dislocation fracture of the first lumbar vertebra and was successfully treated with AE of the lumbar and intercostal arteries.

## Case report

**Figure 1:**
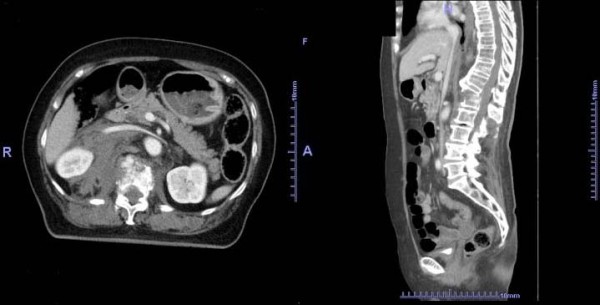
**(a) Initial computed tomography (axial image:left); This CT scan shows a fracture of the first lumbar vertebra along with paravertebral and retroperitoneal hematomas.** (b) Initial computed tomography (sagittal reconstruction:right); This CT scan shows a dislocation fracture (L1).

A 74-year-old woman was injured in a traffic accident. At the scene of the accident, she was found to be alert, and her hemodynamic condition was stable. She arrived at our hospital complaining of lumbago. On arrival, she was conscious and alert, and her other vital signs were as follows: systolic blood pressure, 138 mm Hg; respiratory rate, 16 breaths/min; heart rate, 98 beats/min; and SpO_2_, 100% under 10 L O_2_/min. She had no relevant medical history and was not receiving any medications. Thoracoabdominal computed tomography (CT) with contrast-medium injection was performed; axial and three-dimensional CT scans showed a dislocation fracture of the first lumbar vertebra (type B fracture, according to the Magerl classification) along with paravertebral and retroperitoneal hematomas (Figure 1a, b). No evidence of right renal injury was found on urine analysis and the CT scans. Therefore, we managed the patient conservatively with bed rest.

**Figure 2:**
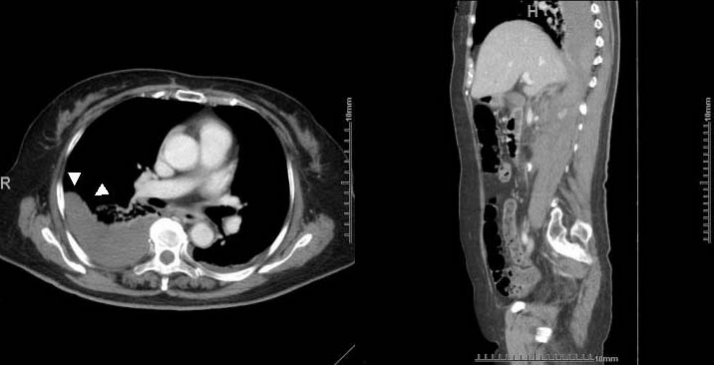
**(a) Thoracic computed tomography performed 3 h after admission (axial image:left); this CT scan shows a right extrapleural hematoma.** One part of the thoracic hematoma shows a D-shaped opacity (Δ). (b) Sagittal-reconstruction computed tomography scan (right); This CT scan shows an enlarged 　　　　　　　　　　　　hematoma, extending from the right retroperitoneal space to the right extrapleural

**Figure 3:**
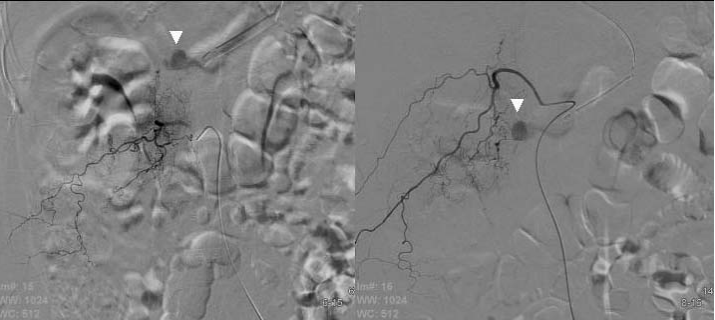
(a) Lumbar (L1) arteriography (left); Extravasation of the contrast medium (Δ) (b) Intercostal (Th12) arteriography (right)；Extravasation of the contrast medium (Δ).

**Figure 4:**
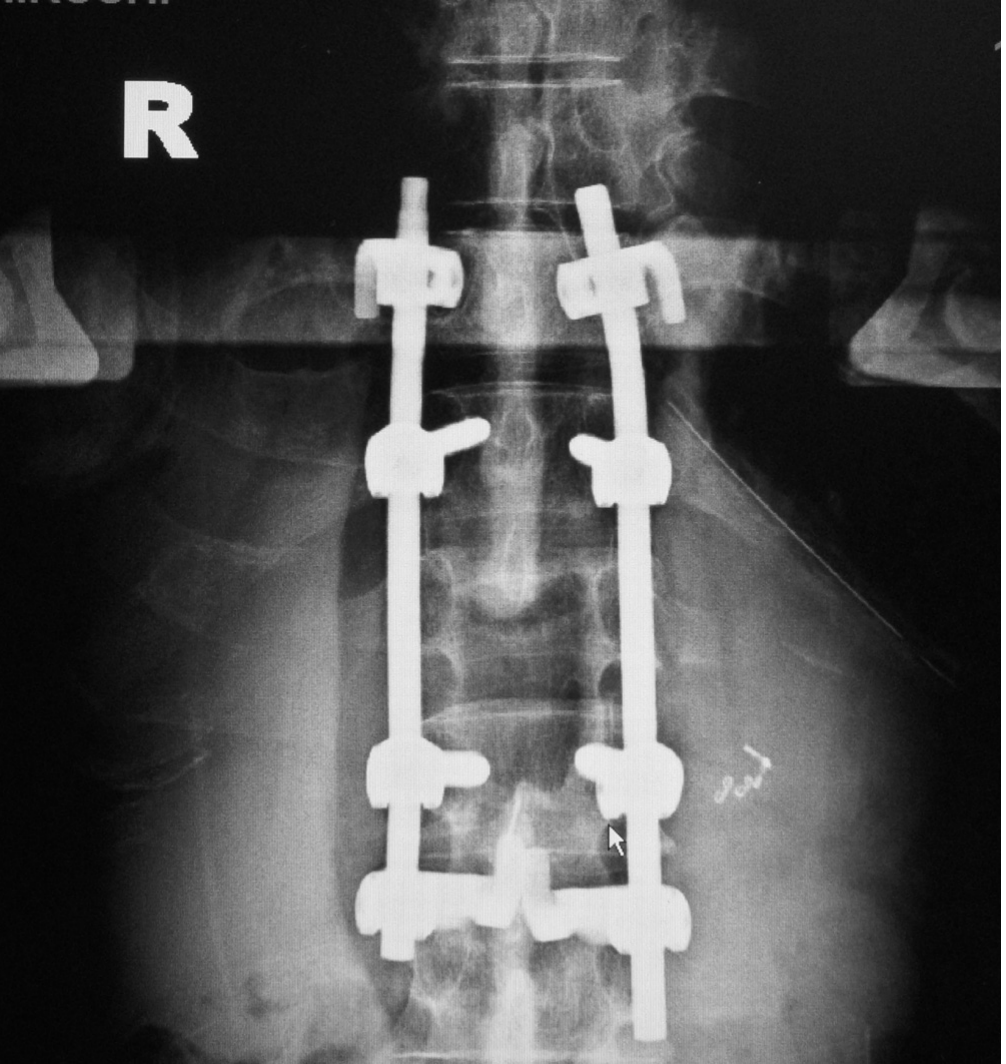
**Postoperative roentgenogram**.

However, 3 h after admission, the patient's blood pressure suddenly decreased from 138/82 mm Hg to 76/40 mm Hg. Her hemodynamic condition stabilized with massive fluid resuscitation, and a repeated thoracoabdominal CT scan with contrast-medium injection was obtained. This CT scan showed enlargement of the right retroperitoneal hematoma with extravasation of the contrast medium and right hemothorax. A sagittal-reconstruction CT scan showed that the hematoma extended from the right retroperitoneal space to the right extrapleural space (Figure 2a, b). Therefore, we concluded that the fluid accumulated in the thoracic cavity was because of an EH and not because of the hemothorax. An angiography was immediately performed to restore hemostasis; a shepherd-hook catheter (4 F, CX catheter A2; Koken, Tokyo, Japan) and a microcatheter (2.4 Fr, Progreat; Terumo, Tokyo, Japan) were used for the angiography. Figure 3 shows the extravasation of the contrast medium from the right intercostal (Th12) and lumbar arteries (L1). After AE with gelatin-sponge particles, the extravasation ceased, and the patient's hemodynamic condition stabilized, without massive fluid resuscitation. The procedure of AE was completed in 30 minutes. The EH reduced in size after AE, and it almost disappeared on the 14^th ^day of hospitalization. On the 16^th ^day of hospitalization, the lumbar spine fracture was successfully repaired (Figure 4), and the patient was kept in the hospital to recuperate.

## Discussion and conclusion

It has been reported that EH is a comparatively rare condition. However, Moheb et al. reported that EH is not uncommon but usually goes unrecognized [1]. There is no appropriate scientific term for hematomas in other abnormal spaces in the chest wall, and these hematomas have been variously referred to as subpleural, epipleural, retropleural, and extrapleural hematomas. Since Moheb et al. reported the nomenclature, classification, and significance of traumatic EHs in 2000 [1], most authors refer to such hematomas as "extrapleural hematomas." EH can be defined as the accumulation of blood in the extrapleural space; however, the site of hemorrhage has not yet been defined. Most of the reported causes of traumatic EH were related to rib fracture, sternum fracture, and thoracic vascular injuries (Table S1, Additional file 1) [3-5]. EH resulting from a hemorrhage site situated outside the chest has not yet been reported. We present the case of a patient with EH caused by an enlarged retroperitoneal hematoma following a fracture of the lumbar spine. The right intercostal and lumber arteries extend over the vertebrae after branching from the aorta. Therefore, we think that the right 12th intercostal artery and the first lumbar artery of our patient were damaged by bone fragments, and that the resultant high-pressure bleeding caused a massive retroperitoneal hematoma and EH.

The typical radiological finding of EH is a D-shaped opacity with its base against the adjacent part of the chest wall; this is because extrapleural bleeding does not result in extravasation of blood into the pleural cavity (cf. epidural hematomas of the head). However, this typical D-shaped opacity was not initially seen in our patient. The basis for our diagnosis of EH was as follows: (1) initial radiological examination revealed no evidence of chest injury; (2) thoracoabdominal CT scans obtained 3 h after admission showed EH along with an enlarged retroperitoneal hematoma; (3) a D-shaped opacity was seen in one part of the thoracic hematoma; and (4) after AE, the thoracic hematoma reduced in size and then disappeared.

Hemorrhage associated with vertebral fractures mainly occurs from the azygos vein, hemiazygos vein, external vertebral venous plexus, and intercostal artery [2]. Bleeding from these vessels leads to the formation of a paravertebral hematoma if the parietal pleura is undamaged. Spontaneous hemostasis usually occurs in these circumstances. A rare case of vertebral fracture presenting with a large life-threatening paravertebral hematoma due to a damaged intercostal artery has been reported [2]. This case was the report in which AE was successfully used for a patient who had developed a life-threatening hematoma following a burst fracture of the thoracic spine [2]. Domenicucci et al. reported the successful treatment of a pseudoaneurysm of the lumber artery that developed after a flexion-distraction injury of the thoracolumbar spine [6]. A few cases of massive hemothorax after thoracic vertebral compression fractures have been reported [7,8]; surgical management was adopted in these cases. Thus, the efficacy of AE in the treatment of hematomas following burst or compression fractures of the spine has not yet been evaluated. AE is less invasive than surgical management, and we believe that AE is effective for the treatment of intractable bleeding following burst or compression fractures of the spine. However, if extravasation of the contrast medium from the intercostal and lumbar arteries into the great anterior radicular artery (artery of Adamkiewicz) is observed on angiography, the method of management should be changed immediately, because embolization of the great anterior radicular artery can lead to spinal ischemia.

## Abbreviations

EH: extrapleural hematoma; AE: arterial embolization; CT: computed tomography.

## Consent

Written informed consent for the publication of this case report and any accompanying images was obtained from the patient. A copy of the consent form is available for review from the Editor-in-Chief of this journal.

## Competing interests

The authors declare that they have no competing interests.

## Authors' contributions

All authors have contributed equally and sufficiently to the conception, design, drafting, and revision of this manuscript.

## Supplementary Material

Additional file 1 **Table S1**. Classification of extrapleural hematomas. Click here for file
