# Supplementary figures and images for: Arterial embolization of an extrapleural hematoma from a dislocated fracture of the lumbar spine: a case report
Source: Scand J Trauma Resusc Emerg Med. 2009 Jun 9;17:27. doi: 10.1186/1757-7241-17-27 (PMC2700073; doi:10.1186/1757-7241-17-27)

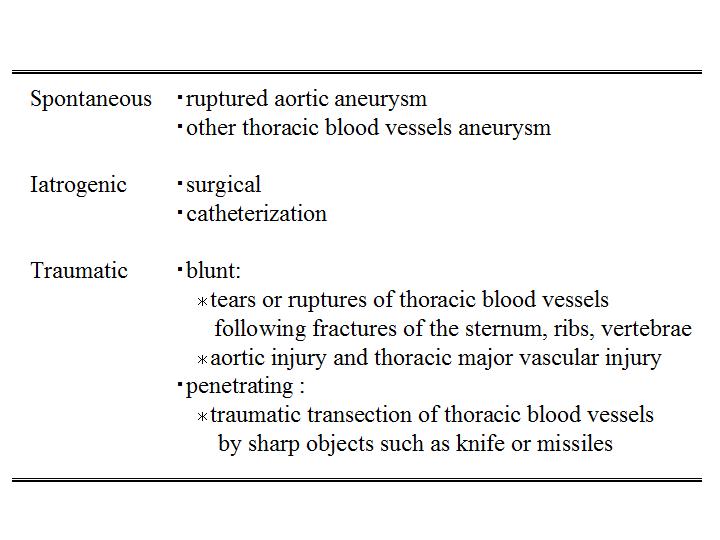

Supplement: Additional file 1 — Table S1. Classification of extrapleural hematomas. [file 1757-7241-17-27-S1.jpeg]
